# Exposures to fine particulate air pollution and respiratory outcomes in adults using two national datasets: a cross-sectional study

**DOI:** 10.1186/1476-069X-11-25

**Published:** 2012-04-10

**Authors:** Keeve E Nachman, Jennifer D Parker

**Affiliations:** 1 Department of Environmental Health Sciences, Johns Hopkins Bloomberg School of Public Health, Baltimore, MD, USA; 2 National Center for Health Statistics, Centers for Disease Control and Prevention, Hyattsville, MD, USA

**Keywords:** Particulate matter, Asthma, Sinusitis, Air pollution, National Health Interview Survey (NHIS)

## Abstract

**Background:**

Relationships between chronic exposures to air pollution and respiratory health outcomes have yet to be clearly articulated for adults. Recent data from nationally representative surveys suggest increasing disparity by race/ethnicity regarding asthma-related morbidity and mortality. The objectives of this study are to evaluate the relationship between annual average ambient fine particulate matter (PM_2.5_) concentrations and respiratory outcomes for adults using modeled air pollution and health outcome data and to examine PM_2.5 _sensitivity across race/ethnicity.

**Methods:**

Respondents from the 2002-2005 National Health Interview Survey (NHIS) were linked to annual kriged PM_2.5 _data from the USEPA AirData system. Logistic regression was employed to investigate increases in ambient PM_2.5 _concentrations and self-reported prevalence of respiratory outcomes including asthma, sinusitis and chronic bronchitis. Models included health, behavioral, demographic and resource-related covariates. Stratified analyses were conducted by race/ethnicity.

**Results:**

Of nearly 110,000 adult respondents, approximately 8,000 and 4,000 reported current asthma and recent attacks, respectively. Overall, odds ratios (OR) for current asthma (0.97 (95% Confidence Interval: 0.87-1.07)) and recent attacks (0.90 (0.78-1.03)) did not suggest an association with a 10 μg/m^3 ^increase in PM_2.5_. Stratified analyses revealed significant associations for non-Hispanic blacks [OR = 1.73 (1.17-2.56) for current asthma and OR = 1.76 (1.07-2.91) for recent attacks] but not for Hispanics and non-Hispanic whites. Significant associations were observed overall (1.18 (1.08-1.30)) and in non-Hispanic whites (1.31 (1.18-1.46)) for sinusitis, but not for chronic bronchitis.

**Conclusions:**

Non-Hispanic blacks may be at increased sensitivity of asthma outcomes from PM_2.5 _exposure. Increased chronic PM_2.5 _exposures in adults may contribute to population sinusitis burdens.

## Background

Relationships between exposure to particulate air pollution and a variety of adverse effects, including cardiovascular and respiratory diseases, birth outcomes, genetic polymorphisms, as well as mortality and life expectancy have been studied [1-8]. A number of studies have investigated the influence of exposure to particulate matter on development of respiratory outcomes, though the majority focus on children [9-13]; a limited number of published reports exist documenting of the effects of chronic exposures on non-cancer respiratory outcomes in adults [14-17].

National prevalence data for several respiratory conditions are available from the National Center for Health Statistics (NCHS) of the Centers for Disease Control and Prevention (CDC), who have estimated that as of 2007, approximately 7, 11 and 3% of non-institutionalized adults reported current asthma, recent sinusitis and chronic bronchitis, respectively [18].

The burden of asthma has been shown to be disproportionately distributed across race/ethnic groups [19]. Much research has evaluated this differential in children [20-23], though relatively fewer investigations have focused on adults. Results from nationally-representative surveys suggest relatively smaller differences in asthma burden by race/ethnicity among adults as compared to children [19], though recent evidence from the National Hospital Discharge Survey and National Vital Statistics System has indicated that disparities in asthma-related hospitalization and mortality may be increasing over time [24,25].

Variations in the burden of sinusitis and chronic bronchitis across population groups have been documented in the 2007 National Health Interview Survey (NHIS) [18]. A recently published summary of indicators measured in the NHIS reported that, as compared to non-Hispanic whites and blacks, Hispanic adults were less likely to have been told they had sinusitis or chronic bronchitis in the last twelve months. Estimates further stratified by sex indicated recent sinusitis and chronic bronchitis diagnoses were most common among non-Hispanic black and white women.

It has been suggested that differential exposures to environmental pollutants may contribute to race/ethnic disparities in health outcomes. Research has found dissimilar distributions of exposure to fine particulate matter across race/ethnicity [26-28]. In addition, increased attention is being paid to the role of racial residential segregation in disproportionate exposures to environmental hazards (such as particulate air pollution) across race/ethnicity [29,30], especially among African Americans [31].

A limited number of studies have examined the potential for race/ethnicity to act as an effect modifier on the relationship between exposure to ambient air pollutants and selected health outcomes. The impact of fine particulate matter exposure on birth weight was found to be differential across race [1]. An examination of the association between nitrogen dioxide exposure on asthma-related hospitalizations in children found race/ethnicity to modify the relationship, even after controlling for health insurance status [32]. While few in number, these studies suggest that race/ethnicity may influence the relationship between exposure to particulate matter and respiratory outcomes.

Evaluating the role of air pollutant exposure in respiratory disease across racial/ethnic groups at the national scale can be facilitated by harnessing the utility of diverse data systems. Integration of data systems initially created for differing purposes is a critical component of the environmental public health tracking initiative [33] that has evolved out of recommendations from the 2000 Pew Environmental Health Commission [34]. A 2006 symposium organized by the USEPA and CDC championed the linkage of air pollution and national health survey data for the purpose of epidemiological investigations; participants identified important data gaps, suggestions for improvements in design and collection of air quality and health data, and other critical considerations [35].

Recent efforts to link ambient monitoring data to health data, in attempts to reduce potential misclassification of exposures, have moved beyond city and county-based measures to employ distance-based metrics [36,37], such as assignment of annual pollution exposure concentrations from the nearest monitor within a specified radial distance from the respondent, or taking the mean (or distance-weighted mean) of all monitored annual average concentrations within that radius. One of the primary shortcomings of using distance-based assignment methods is the inability to assign exposures to respondents for whom monitoring data are not available within the specified radial distance or for whom county-level monitoring data are not collected. Given that monitors are likely to be placed in areas expected to be impacted by air pollution or in more populated areas, inclusion of subjects in epidemiologic investigations is likely to differentially exclude persons living in more rural settings [36].

Since personal exposures to air pollution are not measured as part of the National Center for Health Statistics (NCHS) surveys, previous investigations using these health data have employed distance- or metropolitan statistical area (MSA)-based measures of ambient pollutant concentrations to serve as surrogate measures of exposure [36,38-43]. Geospatial prediction methods such as kriging are becoming more common in environmental research [44], and allow for the estimation of ambient concentrations at unsampled locations. Predictions are made as a function of the spatial autocorrelation of the data, and allow for an estimation of the prediction variance or error in interpolated exposures. A key advantage of using spatial interpolation methods to link ambient pollution data to nationally-administered health surveys is the ability to estimate ambient exposure concentrations for respondents who were not able to be assigned exposures using distance-based methods. Assigning exposures to these respondents allows for a larger study population and may reduce concern over its representativeness by including more persons outside of urban areas. Given the tendency to place monitors in areas with higher concentrations of air pollution, using spatial interpolation will likely result in inclusion of more "control" subjects, or persons exposed to lower ambient pollutant concentrations. Interpolation-based exposure assignment may also reduce the clustering of exposure estimates that can result from using MSA- or county-based averages to assign exposures to study subjects.

The objective of this study is to evaluate the relationship between chronic exposure to fine particulate matter on the prevalence of adverse respiratory outcomes for adults using modeled air pollution data from the EPA AirData system and health outcome data from the National Health Interview Survey (NHIS) geocoded to respondent locations (explained below). In addition, this study evaluates whether these relationships are the same for Hispanic and Non-Hispanic black and white persons. Given the nationally-representative nature of the employed datasets and the diverse study population, interpretation of these results can be informative in the policy setting and for guiding further investigation.

## Methods

### Air monitoring data and criteria for selection of monitors

Annual data for PM_2.5 _for the years 2002-5 were downloaded from the USEPA AirData website [45]. Relevant fields used for model predictions of ambient concentrations using monitoring data were derived from the Annual Summary, Sites, and Monitor tables available from AirData. Criteria were established for the selection of monitor values for inclusion in the kriging interpolation process. Annual arithmetic mean concentrations for PM_2.5 _at each monitor were downloaded from the AirData website for each year between 2002 - 2005. Separate interpolations were performed for each year; for a monitor to be used in interpolation for a given year, the monitor must have reported an annual arithmetic mean for that year. Only monitors from the contiguous 48 states were included in the study, and monitors with missing locational information (latitude or longitude) were excluded.

### Estimation and assignment of exposure from air monitoring data

The AirData system provides locations of monitoring sites in latitude and longitude format. Within the AirData system, the USEPA does not use the same reference datum to assign geographic locations for its monitoring sites. For each monitoring site in the database, one of three reference datums (NAD23, NAD84, and WSG84) was used to assign latitude and longitude data to monitoring sites, except for in some cases, in which the datum used for assignment was not listed and therefore is unknown. An earlier evaluation of potential miscalculations arising from unspecified or incorrectly specified datum found that at the national scale, impacts on distance calculations would be negligible [39].

Monitor locations were converted from a geographic (spherical) coordinate system into a projected (planar) coordinate system to facilitate kriging. Locations were initially projected using the WGS84 projection system in the ArcView GIS version 9.2 software package, and coordinates were re-projected on the North American Equal Albers Conic projection in (X,Y) format (in meters).

Once the data were re-projected, empirical semivariograms using the classical estimator were plotted using the geoR package [46] in the R computing environment [47]. Theoretical semivariograms were plotted over the empirical semivariograms to derive starting parameters for the final semivariogram model estimation. For each year of monitoring data, restricted maximum likelihood estimation was used to evaluate the fit of five models (exponential, spherical, circular, matèrn and cubic correlation functions). Model selection was performed on the basis of comparison of Akaike's Information Criterion (AIC). Subsequent to model selection, an evaluation of predictions was performed using this leave-one-out method, where a measured data point was dropped, and the remaining data points were used to predict the data value at that location. This process, repeated for each measured data point, is known as cross-validation.

### National health interview survey

We combined NHIS data for 2002-2005 for this analysis. The NHIS is a large nationally representative survey of the civilian non-institutionalized population of the United States (information available at http://www.cdc.gov/nchs/nhis.htm) that has been conducted since 1957, although the survey design and questionnaire have changed over time. Very briefly, the NHIS is a cross-sectional household interview survey conducted continuously throughout the year. For these survey years, after state-level stratification, the first stage of its multistage probability design consisted of a sample of 358 primary sampling units (PSUs) drawn from approximately 1,900 geographically defined PSUs. PSUs are counties or groups of counties, or a metropolitan statistical area. Within a PSU second-stage units are drawn (segments) and within each segment a sample of occupied households are selected for interview. Black and Hispanic populations were over-sampled during these years. The probabilities of selection, along with adjustments for nonresponse and post-stratification, are reflected in the sample weights [48]. Additional information is available at ftp://ftp.cdc.gov/pub/Health_Statistics/NCHS/Dataset_Documentation/NHIS/2005/srvydesc.pdf[49].

In 2002-2005, about 35,000 households were sampled each year. In addition to the core family questionnaire that is asked of each family member, a sample adult is selected for additional questions on health and health care [49]. Response rates were generally high. During these data years, information was provided for over 90% of adults selected for the sample adult questionnaire; multiplied by the sample family response rates of 85% to 90%, the unconditional response rate for the sample adult is about 80%.

We used restricted-use NHIS files geocoded to Census block and block-group. These files are available through the NCHS Research Data Center (RDC) (information available at http://www.cdc.gov/nchs/r&d/rdc.htm). Population-weighted census block-group centroids were used as respondent locations.

There are 124,375 adults 18 years of age or older with information for the sample adult questions in the 2002-2005 NHIS. The geographical information was missing for 922 survey respondents, and these persons were excluded from analyses. Those residing within the 48 contiguous states at the time of the interview were included in the analyses. Of these, missing data for one or more of the NHIS variables described below or one or more respiratory conditions resulted in the inclusion of between 109,343 - 109,485 respondents for each outcome.

### Spatial interpolation and data linkage

In the NCHS Research Data Center, Census block-group population-weighted centroid locations of NHIS respondents, described above, were used as prediction locations for estimation of annual average PM_2.5 _exposure concentrations and associated prediction variances. The ordinary kriging method was used to develop predictions and krige prediction variances for each respondent. Weighted predictions of annual average PM_2.5 _concentrations and corresponding prediction variances were generated for each respondent using the fitted semivariogram models corresponding to the NHIS interview year. Annual average PM_2.5 _concentrations were assigned as a continuous variable for exposure measures for survey respondents. The resulting dataset of assigned pollution measures and variances was merged with the dataset for NHIS survey respondents (including outcomes and covariates, described below) to facilitate analyses.

### Respiratory health outcomes

Answers to three questions from the NHIS sample adult questionnaire about asthma and additional questions about sinusitis and chronic bronchitis were used as outcomes in the study. The prevalence of chronic bronchitis and sinusitis were obtained by asking respondents whether during the past 12 months they had been told by a doctor or other health professional that they had these conditions.

To be eligible to provide answers to the questions about asthma, respondents first had to answer affirmatively to having been told by a physician or other health professional that they had asthma. The three follow-up questions queried respondents as to whether they still had asthma, had an episode of asthma or an asthma attack within the last 12 months, or had visited the emergency room or urgent care center due to asthma within the last 12 months. To compare persons with answers for these questions with persons not reporting having asthma, additional variables were created for each of these three measures by combining responses from people providing non-missing values for these measures with persons never reporting having asthma (coded as responding "no" to these questions).

### Covariates

The potential influences of other factors on respiratory outcomes were assessed in the analyses. Respondent race and ethnicity were categorized as Hispanic, non-Hispanic black, non-Hispanic white, and other non-Hispanic race groups. Early exploratory analyses suggested the possibility of heterogeneity of the effect of annual average ambient PM_2.5 _concentrations across race/ethnic strata on respiratory outcomes. Given this, in combination with evidence of a differential in asthma prevalence across race/ethnicity, this variable was examined both as a potential confounding factor as well for purposes of stratification to determine whether air pollution has differential effects on respiratory health outcomes by race/ethnicity.

Possible health-related covariates included sex, age, body mass index (BMI), smoking status, and exercise status. Age was divided into categories starting with respondents' ages 18 to 30 and continuing with ten year intervals up to age 60. A final category was used to represent respondents ages 61 and above. BMI was treated categorically, with BMI < 25 representing normal or underweight, 25 ≤ BMI < 30 representing overweight, and BMI ≥ 30 representing obese. Respondents were characterized as never smokers, former smokers, or current smokers. Exercise status was treated as a binary variable, representing either some or no reported exercise.

Demographic covariates (race and ethnicity, education, and urbanicity) were also examined. Education was treated as a binary variable, representing less than twelve years of education versus twelve or more years of education, regardless of degrees attained; the latter group also included respondents with a GED. The 2006 NCHS Urban-rural Classification Scheme for Counties [50] assigns an urbanicity rating for the 3,141 U.S. counties and county-equivalents. The classification scheme for the rating uses six levels for classification: large central metropolitan, large fringe metropolitan, medium metropolitan, small metropolitan, micropolitan and non-core. The six-level urbanicity rating was initially assigned to all survey respondents based on residential location; for analytical purposes, respondents were subsequently divided into one of two groups. The first group consisted of persons with residences in areas described as large central metropolitan, large fringe metropolitan and medium metropolitan; the other included persons living in areas described as small metropolitan, micropolitan, or non-core. In sensitivity analyses, a more detailed categorization was used.

Covariates related to resource availability and access to care were examined. The ratio of the respondent's family income to the official poverty threshold was categorized into four levels of income as a percent of poverty: less than 100%, 100-199%, 200-399%, and 400% or more. Multiply imputed family income values from NCHS imputed income files [51] were used because income data were missing for many respondents. Respondents were classified by health insurance status as having private insurance, Medicaid, another type of insurance (including Medicare), or no health insurance; respondents with multiple coverage were assigned to one category using the hierarchy listed above.

### Statistical analyses

Logistic regression was used to evaluate the relationship between 10 μg/m^3 ^increases in annual average ambient PM_2.5 _concentrations and respiratory outcomes, controlling for the potentially confounding effects of health and socioeconomic covariates described above. Stratified models were fit to determine whether air pollution had differential effects on respiratory health outcomes by race/ethnicity.

We conducted sensitivity analyses to examine the robustness of our primary findings to levels of urbanicity and insurance status as markers of data consistency. Urbanicity was chosen for further analysis due to the possible compositional differences in PM_2.5 _between more urban and less urban areas [52-54], while health insurance was chosen for further examination due to the possible reporting differences in respiratory outcomes for adults with and without insurance. For these sensitivity analyses, interaction terms between PM_2.5 _and urbanization and health insurance were examined in logistic models for the overall sample and for race/ethnicity groups.

SUDAAN software was used in the regression analysis and tabulation to control for the complex sampling design of the NHIS. All estimates were calculated using the survey weights unless otherwise specified. For NHIS data post-1997, multiple imputations were performed for the family income data, which is used in the computation of poverty level. Five sets of imputed values were created and available on the NHIS public use data files to allow for the assessment of variability caused by imputation. See Schenker et al. (2006) [55] for detail on the imputation methodology and analytic statistical methods.

## Results

### Monitor selection and exposure estimation

**Figure 1:**
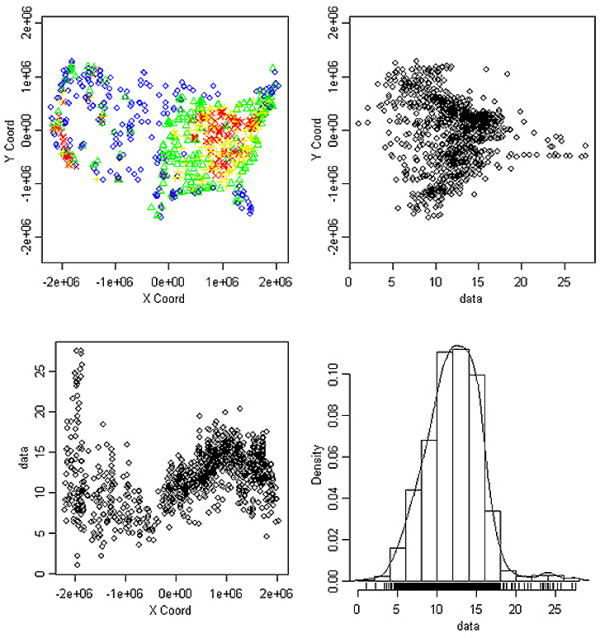
**Data presented are from 2002 EPA monitors**. (A) EPA monitoring sites, color coded for annual average PM_2.5 _concentration by quartile. (B) Scatterplot of the annual average PM_2.5 _concentrations (presented in μg/m^3^) plotted against the Y-coordinates of monitor locations. (C) Scatterplot of the annual average PM_2.5 _concentrations (presented in μg/m^3^) plotted against the X-coordinates of monitor locations. (D) Histogram of the annual average PM_2.5 _concentrations (presented in μg/m^3^). For (A), blue circles denote 1st quartile of PM_2.5 _concentrations, green triangles denote 2nd quartile, yellow plus signs denote 3rd quartile, and red Xs denote 4th quartile.

Application of the specified monitoring criteria to AQS annual monitoring data resulted in the inclusion of 1125, 1111, 1074 and 1051 monitors for each year from 2002 to 2005, respectively. Figure 1 uses 2002 AQS monitoring data to visually demonstrate the spatial distribution of annual average concentrations at USEPA monitoring sites. For all four years of data, as assessed by the AIC, the exponential semivariogram model best fit the data. Results of the leave-one-out cross-validation indicated that mean prediction errors for each year were less than 0.1 μg/m^3^, and interquartile ranges of errors ranged from 1.01 to 1.70, indicating that for re-estimation of actual data, predictions tended to be within 1 μg/m^3 ^of the actual value.

### Study population and results of statistical analyses

Table 1 summarizes study population characteristics for the overall population and for respondents in the top quartile of pollution concentrations by race/ethnicity, including prevalence of health outcomes as well as health-related, demographic, and socioeconomic factors. Rates for current asthma, sinusitis, and chronic bronchitis from the study population were very similar to rates reported by NCHS for 2005 (7%, 13% and 4%, respectively) [56]. Table 2 compares summary statistics of the USEPA monitoring data with those of the kriged predictions at NHIS respondent locations. Results from the primary logistic regression analyses are provided in Tables 3 and 4.

**Table 1:** Percent^1 ^distribution of study population characteristics, overall and among those with highest quartile of exposure, by race/ethnicity

	All^2^	Hispanic	Non-Hispanic Black	Non-Hispanic White
	**N = 109,485, 100%**	**N = 16,942, 10.5%^1^**	**N = 14,351, 10.7%^1^**	**73,495, 73.9%^1^**

Health outcome	Percent^1^	Percent^1^	Percent^1^	Percent^1^

Reported asthma attack in the last year	3.7	3.0	4.1	3.8

Still has asthma	7.0	5.1	8.0	7.1

Asthma ER visit in the last year	1.0	1.2	1.9	0.8

Sinusitis in the last year	14.7	8.6	14.4	16.0

Chronic bronchitis in the last year	4.3	2.7	4.4	4.6

High PM2.5 exposure				

Top quartile	23.3	30.4	28.5	21.4

Sex				

Female	52.6	50.7	56.3	52.4

Age group				

18 to 30	22.1	32.1	26.1	19.6

31 to 40	19.1	25.1	21.0	17.8

41 to 50	20.7	18.7	21.7	20.9

51 to 60	16.0	11.2	14.3	17.1

61+	22.0	12.9	16.8	24.6

BMI classification				

Normal	39.2	32.8	29.8	40.3

Overweight	34.3	37.6	33.9	34.4

Obese	26.5	29.7	36.3	25.3

Smoking status				

Current smoker	20.3	15.7	20.2	21.2

Former smoker	22.8	14.5	14.8	25.7

Never smoker	56.8	69.8	65.0	53.1

Exercise				

None	35.6	47.8	45.9	32.6

Some	64.4	52.3	54.1	67.4

Education				

Less than 12 years of education	13.2	36.3	17.6	9.4

12 or more years of education	86.8	63.7	82.4	90.6

Urbanicity^3^				

Large central metro	27.0	52.8	42.8	20.2

Large fringe metro	24.9	17.9	21.7	26.3

Medium metro	21.4	17.0	17.6	22.6

Small metro	10.1	5.5	6.3	11.6

Less urban	16.7	6.8	11.6	19.3

Percent poverty ratio				

Less than 100%	10.7	20.5	20.3	7.7

100- < 200%	16.8	27.3	22.1	14.5

200- < 400%	31.4	31.0	31.0	31.8

400% or greater	41.1	21.3	26.6	46.0

Health Insurance				

Private	76.0	55.2	62.6	81.0

Medicaid	7.5	15.4	16.0	5.0

Other	8.0	6.7	10.3	8.0

Uninsured	8.5	22.8	11.1	6.0

^1^All percents estimated using survey weights

^2^Includes adults who reported other race/ethnicity groups

^3^Described in text

**Table 2:** Summary statistics for EPA monitoring data and kriged predictions at NHIS survey respondent locations, overall and by race/ethnicity

	Monitoring data^1 ^(μg/m^3 ^PM_2.5_)			Predictions (μg/m^3 ^PM_2.5_)	
	
		All^2^	Hispanic	Non-Hispanic White	Non-Hispanic Black
Min	1.0	4.8	5.2	4.8	5.8

1st quartile	9.8	10.3	9.5	10.3	11.9

Median	12.2	12.6	12.1	12.4	13.4

Mean	12.1	12.4	12.5	12.2	13.2

3rd quartile	14.4	14.4	14.9	14.3	14.7

Max	27.5	24.7	24.7	24.7	24.6

^1^Summary statistics presented are for 2002 monitoring data

^2^Includes adults who reported other race/ethnicity groups

**Table 3:** Adjusted^1 ^odds ratios (95% confidence intervals) for 10 μg/m^3 ^increase in ambient PM_2.5 _concentration and asthma outcomes, overall and stratified by race/ethnicity

	Asthma attack in past year	Still has asthma
**Race/ethnicity**	**AOR (95% CI)**	

All^2^	0.90 (0.78 - 1.03)	0.97 (0.87 - 1.07)

Hispanic	0.99 (0.73 - 1.34)	1.10 (0.85 - 1.43)

Non-Hispanic White	0.85 (0.72 - 1.01)	0.92 (0.81 - 1.04)

Non-Hispanic Black	1.76 (1.07 - 2.91)*	1.73 (1.17 - 2.56)**

* *p *< 0.05, ** *p *< 0.01

^1^Adjusted for sex, age group, smoking status, urbanicity, health insurance type, education, income, body mass index and exercise. Models for All include adjustment for race/ethnicity

^2^Included adults who reported other race/ethnicity groups

**Table 4:** Adjusted^1 ^odds ratios (95% confidence intervals) for 10 μg/m^3 ^increase in ambient PM_2.5 _concentration and other respiratory outcomes, overall and stratified by race/ethnicity

	Sinusitis	Chronic bronchitis
**Race/ethnicity**	**AOR (95% CI)**	

All^2^	1.18 (1.08 - 1.30)**	1.08 (0.94 - 1.24)

Hispanic	0.76 (0.62 - 0.94)*	0.88 (0.67 - 1.16)

Non-Hispanic White	1.31 (1.18 - 1.46)**	1.10 (0.93 - 1.29)

Non-Hispanic Black	1.17 (0.91 - 1.50)	1.50 (0.96 - 2.36)

*p < 0.05, ** *p *< 0.01

^1^Adjusted for sex, age group, smoking status, urbanicity, health insurance type, education, income, body mass index and exercise. Models for All include adjustment for race/ethnicity.

^2^Included adults who reported other race/ethnicity groups.

A 10 μg/m^3 ^increase in estimated ambient PM_2.5 _concentration was not associated with asthma overall or for Hispanic and non-Hispanic white adults. However, among non-Hispanic black adults, we found a significant association between PM_2.5 _and asthma attacks within the last 12 months and reporting still having asthma (Table 3). We found no association between an asthma emergency room or urgent care visit in the prior 12 months and PM_2.5_, either overall or for race/ethnicity subgroups (not shown).

Chronic bronchitis was not significantly related to PM_2.5 _either overall or for subgroups defined by race/ethnicity; the odds ratio for non-Hispanic black adults was elevated but not statistically significant (Table 4). In contrast, sinusitis was significantly related to level of PM_2.5 _overall and among non-Hispanic white adults, while slightly elevated but not statistically significantly for non-Hispanic black adults (Table 4). Increased PM_2.5 _was statistically significantly negatively associated with sinusitis for Hispanic adults (Table 4).

### Results of sensitivity analyses

For the most part, no consistent patterns were observed by level of urbanicity. However, we found evidence that the relationship between PM_2.5 _and both asthma outcomes, still having asthma and asthma attack, among non-Hispanic black adults is most robust in the large central metro areas (still has asthma AOR = 1.94, 95% CI 1.17-3.22; asthma attack AOR = 1.93, 95% CI 1.00-3.71) and elevated, but not significant, in medium metro areas (still has asthma AOR = 2.22, 95% CI 0.92-5.41; asthma attack AOR = 2.52, 95% CI 0.49-13.1) compared to other areas. In contrast, the relationship between PM_2.5 _and sinusitis, overall and among non-Hispanic white adults, may be stronger in less urban compared to more urban areas (not shown). We found no consistent impact of health insurance coverage on the relationship between PM_2.5 _and respiratory outcomes (not shown).

## Discussion

The purpose of this research was to investigate the potential relationship between annual estimates of ambient concentrations of fine particulate matter (as reported by USEPA Airdata) and the prevalence of self-reported respiratory conditions from adult respondents 18 years of age and older from the NHIS. Analyses using the general study population did not find associations between increases in fine particulate matter and self-reported current asthma status or recent asthma attacks, though recent report of sinusitis was found to be significantly associated with increases in ambient PM_2.5 _concentrations. Stratified analyses performed as part of this research suggests that, in non-Hispanic black persons, especially those living in urban areas, asthma-related morbidity is associated with higher concentrations of fine particulate matter averaged over a year.

It is difficult to determine whether the observed differences across racial/ethnic strata are due to difficulties in classification of exposures and outcomes, physiologic variations across race/ethnicity in response to particulate matter, other confounding factors that were uncontrollable in the present study, or a combination of these. Even with the ability of the NHIS to account for a variety of pertinent covariates, many of these influential factors were only able to be addressed at a crude level in the current study.

It is unavoidable that use of ambient monitoring data to characterize exposure to air pollution and self-reported morbidity data will result in measurement error that introduces uncertainties into the interpretation of findings. Data from ambient monitors are not an ideal surrogate for personal exposure measurements, as ambient air is only useful in prediction of air quality in some microenvironments. Despite this shortcoming, relative differences in ambient exposures across persons may be informative for comparisons with health outcomes. Further, ambient concentrations of PM have been found to be highly correlated to personal exposures to ambient-generated PM [57]. Consequently, residence-based non-ambient generated sources of PM (such as environmental tobacco smoke and cooking) and occupational sources (such as diesel exhaust) that we were unable to account for in this study are likely to result in non-differential misclassifications of total personal exposure to PM, ultimately biasing associations towards the null. Further, use of annual average PM_2.5 _concentrations may mask important shorter-term variations in true exposure profiles relevant to respiratory morbidity. While USEPA PM_2.5 _data are available at finer temporal resolution, the temporal nature and frequency of collection of outcome data from NHIS precluded evaluation of potential relationships at a finer temporal scale.

Our sensitivity analyses by urbanicity were suggestive of different effects across locations, but were not definitive. Fine particulate matter is a heterogeneous mixture that tends to vary in constituency over time and across space [58]. Given the nature of the exposure and outcome measurements used in this study, the ability to evaluate the influence of temporal changes in (or seasonality of) PM_2.5 _species was limited. It has been shown in a variety of locales that the composition of fine particulate matter varies between urban and rural settings [52-54], potentially as a function of particulate sources [59]. The composition of fine particulate matter in urban settings has been shown to be higher in elemental carbon (EC) content [54], an indicator of diesel exhaust [60] that has been shown to adversely influence indicators of respiratory capacity in the general population [61] and in asthmatics [62]. Variability in particulate composition across these settings, especially of constituents known to exacerbate asthma, may help explain our finding of stronger relationships between PM_2.5 _exposure and reporting asthma outcomes in urban settings. There is also suggestive evidence that urban residence, independent of race and income, predicts asthma morbidity [63,64].

The outcomes data used in this assessment also require careful consideration. Self-reported health prevalence data are subject to information biases that may be differential across a variety of factors. Interpretation of these data as prevalence of health outcomes is complicated by the fact that some survey questions query whether a health professional has informed the respondent that he or she has the outcome of interest. Such a query inherently makes assumptions about the respondent's resources to acquire health care that may be faulty and ultimately lead to an underreporting of health outcomes. Further, diagnosis-related difficulties for asthma stemming from the lack of a definitive clinical test and issues with consistency across health practitioners to non-specific case definitions may result in inaccurate identification of cases [19,65]. Beyond diagnosis-related issues are concerns related to specificity of the outcomes. In particular, the asthma attack query requires subjective judgment. Is an exacerbation of symptoms that is quickly remedied by use of asthma medication considered to be an attack? Or is there some symptomatic severity threshold that constitutes an asthma attack? Misclassification of outcomes may introduce bias into the assessment of association, though the direction of the bias (if any) is difficult to predict.

Given the dependence of the outcomes on physician diagnoses, analyses were performed to determine model sensitivity to stratification by health insurance type; these analyses found stratum-specific effect estimate magnitudes to be stable for both asthma outcomes.

Racial disparities in asthma-related hospitalization and mortality among children have been repeatedly identified in the literature [66], though far fewer evaluations of potential disparities among adults have been published. An analysis of NHIS asthma prevalence data from 1980 to 2004 reported varying disparities in asthma prevalence between blacks and whites by age group, with a five percent difference in children and less than a single percent difference in adults [19]. Hasselkorn et al. (2008) [67] found that, as compared to whites, an increase in asthma control problems among black persons persisted after controlling for factors related to demographics, asthma severity and co-morbidities. Few studies of gene associations have been performed among persons of African ancestry [68], though existing research has suggested variability in genetic pathogenesis of asthma across racial/ethnic groups [22]. However, in a review of racial disparities in asthma prevalence, Wright and Subramanian (2007) argued that the simultaneously growing disparities and increases in asthma prevalence and severity over the past twenty to thirty years are evidence against this variability being the most important factor in asthma pathogenesis, suggesting that genetic shifts capable of these observed differences would be unlikely in such a short time period. Instead, the authors argue that gene-environment interactions are likely strong factors in the observed changes in asthma burden [69]; accordingly, an underlying genetic susceptibility to asthma development may help explain observed differences that were not able to be explained solely by variations in exposures to environmental pollution. Race/ethnic differences in distributions of atopy have been observed [70], and climate-related factors specific to geographic areas have been demonstrated to interact with exposure to air pollution in prediction of asthma and allergic rhinitis [71], suggesting the possible cumulative contribution of allergy and regional climate variation to the observed differences. This assertion may provide support for our finding of increased susceptibility of non-Hispanic black respondents to asthma-related outcomes as a result of higher exposures to PM_2.5_.

One limitation of this study is its lack of ability to draw inferences regarding the relationship between PM exposure and asthma outcomes in Hispanic persons. Research has shown that the Hispanic race/ethnicity group is comprised of persons of varied backgrounds, and that rates of asthma within these subgroups are highly inconsistent [72,73]. The collapsing of these multiple subgroups with different rates of asthma into a single race/ethnic stratum is likely to obscure our ability to capture any potential effect of PM on the studied outcomes.

In addition to PM, nitrogen dioxide, ozone, and sulfur dioxide are criteria pollutants that have also been demonstrated to have an effect on asthmatics [74,75]. The robustness of the USEPA AQS PM_2.5 _monitoring network and the spatially homogenous nature of particulate matter made possible the kriging of annual concentrations to allow for exposure estimations for participants with residences further away from monitored locations. However, the characteristics of the AQS monitoring network size and data collection schedule, as well as the spatial representativeness of monitored concentrations of these other pollutants precluded their estimation at respondent locations and subsequent inclusion as covariates in our models. Similarly, some non-criteria hazardous air pollutants have also been suspected to be influential in the development and exacerbation of asthma [76], but monitoring networks for these toxics are typically more limited than those for criteria pollutants and may not support prediction of exposure for NHIS respondents. It is not possible to predict the influence of the exclusion of these pollutants from our investigation; future study is warranted to delineate the potential joint contributions of ambient air contaminants.

The social environment has been demonstrated to be an influential factor in the exacerbation of asthma and severity of asthma attacks [77], and evidence in adolescents and young adults exists to suggest that stress may alter or induce asthma-related immune response [78-80]. Recent literature has identified the potential for interaction between environmental and social stressors in causing morbidity [81,82]. Chen et al. (2008) [83] evaluated the interaction between exposures to traffic-related air pollution and chronic family stress in exacerbation of asthma, and found that in children exposed to relatively lower levels of air pollution, high levels of chronic family stress increased child- and parent-reported asthma symptoms and reduced clinical measures of respiratory capacity. More recently, researchers have used genomic methods to determine that genes relevant to asthma-related inflammatory mechanisms were overexpressed in children of lower socioeconomic standing [84]. Measures of these factors were unavailable in the NHIS, though an attempt was made to account for this through adjustment for urbanicity, education and poverty ratio. Despite this, it is likely that the effect estimates are influenced by residual confounding and are likely attenuated.

## Conclusions

Using two linked national datasets and stratified analyses, we found compelling evidence in support of the relationship between increases in ambient PM_2.5 _and asthma outcomes in non-Hispanic black adults. In addition, we identified a relationship between increased fine particulate exposure and the development of sinusitis among all adults. Given that non-Hispanic black adults suffer greater morbidity and mortality from asthma than other groups, and consequently attribute greater medical costs from asthma, further investigation is warranted to better explain the apparent racial/ethnic disparity in asthma prevalence.

## Abbreviations

AIC: Akaike Information Criterion; AOR: Adjusted Odds Ratio; AQS: Air Quality System (USEPA database); BMI: Body mass index; EC: Elemental carbon; MSA: Metropolitan statistical area; NCHS: National Center for Health Statistics; NHIS: National Health Interview Survey; PM_2.5_: Particulate matter less than or equal to _2.5 _microns in diameter; PSU: Primary sampling unit; RDC: NCHS Research Data Center; USEPA: United States Environmental Protection Agency.

## Competing interests

The authors declare that they have no competing interests.

## Authors' contributions

KEN was responsible for co-development of concept, cleaning, management, analysis and interpretation of data, and drafting the manuscript. JDP was responsible for co-development of concept, advising on analysis, interpretation and presentation of data, and assistance in drafting manuscript. Both authors read and approved the final manuscript.
